# β-D-Glucan Assay in Diagnosis and Monitoring the Systemic Candidiasis in a Rat Model

**DOI:** 10.5812/jjm.10247

**Published:** 2014-06-01

**Authors:** Hossein Khodadadi, Hossein Mirhendi, Koichi Makimura, Kazuo Satoh, Ladan Karimi, Shahrokh Izadi

**Affiliations:** 1 Department of Medical Parasitology and Mycology, School of Medicine, Shiraz University of Medical Sciences, Shiraz, IR Iran; 2 Department of Medical Parasitology and Mycology, School of Public Health, National Institute of Health Research, Tehran University of Medical Sciences, Tehran, IR Iran; 3 Institute of Medical Mycology, Teikyo University, Tokyo, Japan; 4 Center of Medical Commission and Occupational Medicine, Social Security Organization, Esfahan, IR Iran

**Keywords:** Beta-D-glucan, Systemic candidiasis, Diagnosis

## Abstract

**Background:**

Determination of β-D-Glucan (BDG) in the serum aids to diagnose the invasive fungal infections. The current study evaluated the diagnostic potential value of BDG assay in monitoring the disease in experimental systemic candidiasis in a rat model. The results can provide a useful preliminary data to improve this approach in developing countries.

**Objectives:**

The present study aimed to evaluate β-D-Glucan assay in diagnosis and monitoring the systemic candidiasis in a rat model.

**Materials and Methods:**

Twenty one rats were infected with 10^6^ *Candida albicans* blastospore per rat. Twelve rats were considered as the negative controls (six immunocompromised rats without infection and six intact rats). During a week, every 24 hours the BDG sera level was determined by both Fungitell and Wako kits. To confirm the systemic infection in each rat, the suspensions of their internal organs were cultivated on agar plates and the number of colony forming units (CFU) of *C. albicans* was counted.

**Results:**

All the infected rats were positive with BDG tests. An increasing level of BDG was observed during early days after injection. The cutoff value for discrimination of BDG positive sera was obtained from the negative sera by the Fungitell kit. The sensitivity, specificity, positive and negative predictive values assessed for the Fungitell kit were 95%, 66.6%, 90.47% and 80%, respectively. These criteria for those of Wako were 90%, 83.3%, 94.7% and 71.4%, respectively.

**Conclusions:**

While BDG assay seems to be a sensitive and specific adjunctive tool to diagnose and monitor the experimental systemic candidiasis, it seems that measuring the positive cutoff value in different laboratory conditions is necessary for favorable establishment of these tests.

## 1. Background

Invasive fungal infections (IFIs) have increased in recent decades. Widespread use of broad spectrum antibiotics, immunosuppressive drugs, use of indwelling devices, and increased population of patients undergoing organ transplantation are the major causes of this rise (1, 2). Invasive aspergillosis and candidiasis are the most important and common IFIs in immunocompromised patients. The mortality rate of patients infected with these fungi is high and up to 50% (3, 4). Therefore a non-invasive, rapid, and reliable diagnostic tool, as well as an early and targeted treatment to monitor the disease, and avoid unnecessary use of potentially toxic and costly drugs are critical.

**Figure 1.**
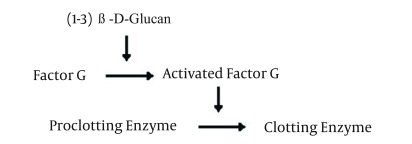
Glucan Effect on *Limulus* Amebocyte Lysate Coagulation Pathway

Conventional culture-based methods such as blood culture variants lack enough sensitivity , therefore in the best conditions they are positive in only less than 50% of candidiasis, and 10% of invasive aspergillosis cases, and it takes several days to achieve the results (5-7). Recently non-culture based methods to detect fungal elements in at-risk patients have been described. Enzyme Immunoassays (EIA) to detect fungal antigens, polymerase chain reaction (PCR) for specific DNA, and assays detecting fungal components and metabolites such as mannan, glucan, or enolase have been reported (6, 8-10). However, even by using these approaches, reliable diagnoses of IFIs are still problematic.

(1-3) Beta-D-Glucan (BDG) is a major structural component of many fungal cell walls excluding Zygomycetes and *Cryptococcus neoformans* which release no or little BDG in the human serum (11, 12). (1-3) Beta-D-Glucan and bacterial endotoxin can activate different coagulation cascades in horseshoe crab amebocyte lysate. Endotoxin specifically activates factor B and C while BDG activates factor G. By removal of factor C from *Limulus* amebocyte lysate, coagulation cascade is activated only by BDG (Figure 1). 

The activation rate of this coagulation cascade can be assessed and quantified by colorimetric or turbidimetric methods. Commercial assay systems to detect BDG apply different reagents derived from different species of horse shoe crabs, therefore these assays have different positive cutoff values (13). The method was established first in 1995 in Japan and then in 2004 in USA and has been recommended as one of the indirect mycological criteria to diagnose invasive fungal infection in the diagnostic guideline published by the European Organization for Research and Treatment of Cancer Invasive Fungal Infections Cooperative Group, and National Institute of Allergy and Infectious Diseases Mycoses Study Group [EORTC/MSG] (14) in 2008. 

It is well documented that presence of BDG in plasma or serum of at-risk patients is a valuable marker for invasive fungal infection (2). Nevertheless, routine application of this test has been problematic in many laboratories and the corresponding kits are approved in limited countries. The current study, in an attempt to evaluate usefulness of BDG assay, assessed the test by two different kits with different methods in two different laboratories based on a rat model of the experimental systemic candidiasis. The results of the experiment can provide useful preliminary data to improve and establish the BDG approaches in Iran and other developing countries.

## 2. Objectives

The present study aimed to evaluate β-D-Glucan assay in diagnosis and monitoring the systemic candidiasis in a rat model.

## 3. Materials and Methods

### 3.1. Animals

This study was performed according to the ethical principles for animal research (National Ethical Framework for Animal Research in Iran). Thirty three 8-12 week-old male rats (Sprague-Dawley), weighing 220 ± 35 g, were used for this study. Immunosuppression and neutropenia were induced in 27 out of 33 rats by intravenous administration of 200 mg/kg body weight of cyclophosphamide (Baxter Oncology GmbH Frankfurt, Germany) four days before inoculation and additional doses of 50 mg/kg on the day of inoculation (15, 16). Six out of 27 immunosuppressed and six intact rats were used as the negative controls without any inoculations.

### 3.2. Preparation of Yeast Cells for Injection

A *Candida albicans* strain isolated from a patient with candidemia was used to prepare microbial suspension. The yeast was sub-cultured on Sabouraud dextrose agar (SDA, Merck KGaA, Darmstadt, Germany) plate for 48 hours at 37˚C and harvested colonies were suspended in 10 mL sterile normal saline solution (NaCl 0.9%) and washed twice. Viable yeast cells in suspension were counted with a hemocytometer and the number of yeast cells adjusted to 1 × 10^6^ per milliliter. To confirm the cell count, tenfold serial dilutions of suspension from 10^6^ to 10^0^ were prepared and 100 microliter of each dilution was spread onto SDA, and colonies were counted after 48 hours at 37˚C. 

### 3.3. Induction of the Experimental Infection in the Rat Model

A one hundred microliters aliquot (1 × 10^5^ cells) of *C. albicans* suspension was injected into the tail vein of 21 rats. During a period of seven days after inoculation, every twenty four hours three rats were anesthetized with chloroform and their cardiac puncture blood was collected in both EDTA free and EDTA coated tubes. Clotted blood was centrifuged for 5 minutes at 1300rpm and the supernatant (serum) was separated and kept in -20˚C until use. The course of infection was evaluated by weighing the rats every day and assessing their weight loss as well as the yeast burden in their internal organs after sacrificing the animals.

### 3.4. Pathological Studies

Brain, heart, kidneys, spleen and liver of each rat were removed and about 0.5 g of each organ was homogenized in 2mL of sterile normal saline. One hundred microliters of blood and the homogenate of rat organs were cultured on SDA plates for 48 hours at 37˚C and then colonies were counted. Each homogenate was also examined microscopically.

Glucan assay: Beta-D-Glucan levels in all sera samples were assessed with two kits (Fungitell and Wako): Fungitell assay (Associates of Cape Cod Inc., Falmouth, Mass, Associates of Cape Cod Inc., Falmouth, Mass, USA) determines levels of BDG in serum or plasma. The detection reagent is a biological cascade based upon modified *Limulus* amebocyte lysate (LAL), an extract of the blood cells of the North American horseshoe crab. The Fungitell reagent is modified to eliminate factor C and, thus bypassing the activated factor B, only reacts to BDG, through the factor G-mediated side of the pathway. This renders the reagent highly specific for BDG and does not react to other polysaccharides, including beta-glucans with different glycosidic linkages.

The assay was fulfilled according to the manufacturer instruction. Briefly, 5 μL of serum or plasma was added to duplicate wells of a 96-well microtiter plate and pretreated with 20 μL of serum treatment reagent (a 1:1 mixture of 0.125 M KOH and 0.6 M KCl) for 10 minutes at 37˚C. Then 25 μL of each standard (6.25 to 100 pg/mL Glucan) was added to each standard well. One hundred microliter of Fungitell reagent (*Limulus* amebocyte lysates with pyrosol as substrate) was added to each sample and standard or blank well. The rate of change of absorbance per minute (ΔOD/ min) at 405 nm (with 490 nm background subtraction) was monitored for 40 minutes at 37˚C by using a THERMO max automated microplate reader (Molecular Devices) equipped with SOFT max PRO software, version 3.1.1. A kinetic standard curve was constructed and BDG concentrations of unknown samples were interpolated from standard curve.

The beta-glucan Wako test (Wako Pure Chemical Industries, Osaka, Japan) also determines levels of BDG in plasma. The mechanism of the assay is the same as Fungitell assay with the difference that this assay uses turbidimetric detection method instead of colorimetric method. The procedure for this assay was conducted according to the manufacturer instruction. Briefly, 100 μL of plasma was added to BDG free tubes containing 900 μL of pretreatment solution (0.2% triton X-100, 0.002% polymyxin B). Tubes were incubated at 80˚C for 10 minutes to neutralize any contaminating endotoxin. After cooling the tubes for 5 minutes, 100 μL of the supernatant of pretreated samples was added and mixed with 100 μL of LAL reagent. In this process, BDG activates the coagulation reaction cascade. Increased turbidity was determined with the kinetic turbidimetric method using Toxinometer MT 251 (Wako Pure Chemical Industries, Wako Pure Chemical Industries, Osaka, Japan). The coagulation time (Tc) was defined as the reaction time required for the transmittance to diminish the threshold values (98% of the initial transmittance). Therefore the higher the level of BDG in the sample, the shorter the coagulation time, and log (BDG) is inversely proportionate with log (Tc). Manufacturer has recommended a cutoff value of 11 pg/mL for this assay. 

In order to validate the precision and repeatability of Fungitell assay, intra-assay (within run assay), and inter-assay (between run assays) were evaluated. To assess inter-assay variabilities the tests of all 30 sera samples of infected and non- infected rats were repeated on three different days. Intra-assay variabilities of the Fungitell assay were assessed by retesting three different serum samples, one serum with lower cutoff range of manufacturer (19.5 pg/mL), one in approximate range of cutoff value (71.88 pg/mL), and one with a higher range than the cutoff value, (646 pg/mL) in quadruplicate in a single assay. Replicate of each sample was placed in different locations within the microtiter plate. The mean and coefficients of variation (CVs) for replicates of the three samples were calculated. To evaluate glucan assay, all 30 sera samples were analyzed in Medical Mycology Laboratory of Tehran University of Medical Sciences (TUMS) with Fungitell kit and were also sent to Teikyo University, Institute of Medical Mycology, Tokyo, Japan (TIMM) to determine BDG concentrations with Wako kit.

## 4. Results

Three rats died during the experiment and after intravenous inoculation with 1 × 10^5^ CFU of *C. albicans*. The remaining 30 rats were sacrificed after cardiac puncture according to the ethical principles for animal research.

### 4.1. Organ Deposition of C. albicans Blastospors

Distribution of yeast in internal organs of rats, according to the colony count of homogenates cultured on SDA, was summarized in Table 1. The severity of infection increased in heart, brain, liver, spleen and kidneys respectively. Only five blood samples cultured on SDA plates showed colonies of yeast having low colony counts, however, all rats showed organ involvement with *C. albicans*.

**Table 1.** Approximate Number of Candida CFU per 100 Microliter of Tissue Homogenates and Blood Cultured on SDA Plates

Rat Number	1	2	3	4	5	6	7	8	9	10	11^a^	12	13	14	15^a^	16^a^	17	18	19	20	21
**Kidney**	10^2^	<10	10^5^	10^1^	105	< 10	10^4^	10^5^	10^5^	10^3^	-	10^3^	10^5^	10^3^	-	-	10^4^	10^5^	10^4^	10^5^	10^5^
**Spleen**	10^1^	<10	10^4^	10^2^	10^3^	< 10	< 10	10^5^	10^5^	10^4^	-	10^3^	10^4^	10^2^	-	-	10^2^	10^4^	10^2^	10^4^	10^4^
**Liver**	< 10	0	10^1^	< 10	10^1^	< 10	10^1^	10^2^	10^3^	10^1^	-	< 10	10^2^	< 10	-	-	10^1^	10^2^	10^1^	10^2^	10^2^
**Heart**	< 10	0	10^3^	< 10	10^2^	0	< 10	10^3^	10^4^	10^1^	-	< 10	10^3^	< 10	-	-	10^2^	10^2^	10^3^	10^4^	10^1^
**Brain**	0	0	10^1^	< 10	0	0	< 10	10^2^	10^2^	< 10	-	10^2^	10^2^	< 10	-	-	0	10^1^	10^1^	10^2^	10^1^
**Blood**	< 10	0	0	0	0	0	< 10	0	0	0	-	0	0	0	-	-	0	< 10	0	< 10	< 10

^a^ Rats number 11, 15 and 16 died during the experiment.

### 4.2. Evaluation of Fungitell and Wako Assay in Rat Serum

Using the predefined cutoff value of 80 pg/mL for Fungitell and 11 pg/mL for Wako, 18 sera samples of the 19 rats with the systemic candidiasis that had high level of BDG with a mean BDG concentration of 738.41 (ranged 28 to 1288 pg/mL) were assessed with Fungitell, and the mean concentration 632.7 (ranged 5 to 3103 pg/mL) was assessed with Wako.

### 4.3. Fungitell Assay Reproducibility

The inter-assay coefficients of variances (CVs) for the three runs were 5.8% to 20.9%, and 32.1%, for the serum samples with BDG concentrations of 19.5 pg/mL, 71.88, and 646, respectively. The mean inter-assay CV for all 30 samples was 19.6%. The intra-assay CVs for the three tested samples were 3.3% and 5.8% for the samples with BDG concentrations of 19.5 and 71.88 pg/mL, respectively. Sample with BDG values of 646 pg/mL had replicate CVs of 11.6%. 

### 4.4. Determination of the BDG Cutoff Value for Fungitell Assay

The serum samples collected from 12 non-infected rats (six immunocompromised rats without infection and six intact rats) were assessed by Fungitell, tested at TUMS, had a mean BDG concentration of 93.4 pg/mL (range 16.8-157 pg/mL) and out of these 12 samples, five serum samples had > 80 pg/mL (84, 87,117, 143,157 pg/mL) BDG concentration. By repeating the assay two more times (inter-assay) similar results were obtained with a mean CV around 8.3% for all of the control samples. Infected rats had high level of BDG with a mean BDG concentration of 738.41 pg/mL. Thus 120 pg/mL was used as positive cutoff value to discriminate BDG positive samples from the negative ones. Using Wako assay non-infected rat sera had a mean BDG concentration 0f 4.2 pg/mL (rang 2.5-27.4). Non-infected rat sera samples did not have BDG concentration >27.4 pg/mL. Intra- and inter-assays were not done on the control sera by this kit. The mean BDG value for the infected rat sera was 632.70 pg/mL (rang 5-3103 pg/mL). Predefined cutoff value of 11 pg/mL was used.

Diagnostic efficiency: With the calculated positive cutoff value > 120 pg/mL for Fungitell assay the sensitivity, specificity, Positive Predictive Value (PPV), and Negative Predictive Value (NPV) of the Fungitell were 95%, 66.6%, 90.47% and 80% respectively (Table 2) and with the positive cutoff value > 11 pg/mL the sensitivity, specificity, PPV, and NPV of the Wako assay were 90%, 83.3%, 94.7% and 71.4% respectively (Table 2).

**Table 2.** Comparing the Efficiency of Fungitell and Wako Kits Based on Newly Calculated Cutoff Values ^a,b^

-	Sensitivity	Specificity	PPV	NPV
**Fungitell**	95	66.6	90.47	80
**Wako**	90	83.3	94.7	71.4

^a^ Abbreviations: NPV; Negative Predictive Value; PPV: Positive Predictive Value

^b^ Data are presented as %.

## 5. Discussion

Measurement of BDG has been recommended in some guidelines from Japan, Europe and USA to diagnose IFIs. The commercial kits used in these countries have different measurement methods (colorimetric or turbidimetric) and cutoff values thus sensitivity, specificity, PPV and NPV reported for each kit and for each experiment have shown large differences; then judgment on positive and negative results could be a laborious work leading to several clinical problems (17). Since a lot of factors could interfere with the results, it is very important that each laboratory sets up the test and calculates its own cutoff values and other diagnostic efficiency criteria. 

The current study evaluated applicability of two BDG assays (Fungitell and Wako) to diagnose and monitor an IFI (systemic candidiasis) in an experimental model in order to use them as adjunctive tools for IFIs diagnosis in patients in Iran. The current experiment used sera of immunocompromised rats inoculated with *C. albicans* as proven IFI case group and sera of intact rats as the control group. Development of IFI (systemic candidiasis) in rats was approved by cultivation of homogenized organ suspensions on SDA and their microscopic examination. According to the results stated in Table 1, deposition of *C.* *albicans* blastospors in Spargue-Dawelly organs was different from those of blastospors in BalB/C and CBA mice reported previously (18), and burden of infection increased in heart, brain, liver, spleen and kidneys respectively.

To evaluate the efficiency of these two assays the current study considered 120 pg/mL as cutoff value for Fungitell assay in the experiments. This cutoff value is higher than those recommended by manufacturer (80 pg/mL). Pezos et al. used 120 pg/mL as positive cutoff value in their experiments, too (12). Using this cutoff value, the sensitivity, specificity, PPV and NPV of Fungitell assays were 95%, 66.6%, 90.47% and 80%, respectively. According to the predefined cutoff value of 11 pg/mL by Wako assay, the sensitivity, specificity, PPV and NPV were 90%, 83.3%, 94.7% and 71.4%, respectively. These results are comparable to the ones published by Yoshida et al. (17), and look reliable. Of course Karageorgopoulos et al. in his Meta-analysis study reported a total sensitivity of 77% and specificity of 85% that shows a lower sensitivity than specificity for BDG assay (19). 

False positive BDG results observed in the current experiment decreased significantly when the cutoff value was corrected to 120 pg/mL. Some interfering conditions such as the dusty environment in Iran could change negative results to false positive. But this assumption should be approved with more studies. Coefficients of variances for inter and intra assays (Mean CVs 19.6% and 6.9% respectively) showed a poor reproducibility performance for Fungitell kit especially on measurement of BDG concentration higher than the cutoff value. In summary, the current study suggests that measuring BDG levels in serum of a patient suspected to IFIs is a helpful and adjunctive tool to diagnose and monitor the disease if the below point is considered as other researchers have noted (19, 20).

A frequency of two tests per week should be performed on each patient. Results of the BDG assay should be interpreted by a companion clinical, radiological and other laboratory criteria for the diagnosis of IFIs. Each laboratory should calculate its own cutoff value and perform quality control programs to qualify the applied kit to reduce false positive and negative results. Proper use of the test depends on the experienced technologist who performs the assay, and maximum reduction of the specimens contamination with gauze, environmental dusts and organic wastes in the lab.
